# Author response to: Enhancing prehabilitation protocols in frail older adults undergoing joint replacement - methodological insights from a pilot randomized controlled trial

**DOI:** 10.1016/j.tjfa.2025.100067

**Published:** 2025-07-11

**Authors:** Alexandra Papaioannou, Ashlee Azizudin, George Ioannidis, Fit-Joints study investigators

**Affiliations:** a Department of Medicine, McMaster University, Hamilton, ON, Canada; b Geras Centre for Aging Research, St. Peter’s Hospital, Hamilton Health Sciences, Hamilton, ON, Canada; c Department of Health Research Methods, Evidence, and Impact, McMaster University, Hamilton, ON, Canada

We thank the authors of the Letter to the Editor for their thoughtful commentary on our manuscript, “*Getting fit for hip and knee replacement: The Fit-Joints multimodal intervention for frail patients with osteoarthritis – a pilot randomized controlled trial”*[1]. We appreciate the recognition of our efforts to advance prehabilitation for frail older adults undergoing total hip or knee arthroplasty and welcome the opportunity to address the important methodological considerations raised.

We agree that pain is a critical domain in the context of frailty and surgical recovery. In our study, we collected self-reported pain outcomes as part of the Oxford Hip Score (OHS), Oxford Knee Score (OKS), and the Geras Fit-Frailty Index, which include validated pain-related subdomains. The OHS and OKS are widely used, validated measures for assessing pain and function in patients undergoing hip and knee arthroplasty, respectively. They have been validated against established pain and function measurements including the 36-Item Short Form Survey (SF-36), Stanford Health Assessment Questionnaire, and American Knee Society score[2]. There were clinically relevant changes in the Oxford Knee Score at 6-weeks postoperative (9.11 [95 % CI: −2.66 – 20.87]) and the Fit-Frailty Index (−0.04 [95 % CI: −0.10 – 0.01]) at 6 months postoperative. Additionally, we reported on pain-related adverse events. There were 83 adverse events reported across 44 participants. Pain was one of the most common adverse events reported (*n* = 21 [25 %]). Specifically, 10/21 (48 %) of these events were reported as pain that occurred during exercise (8 preoperatively, 2 postoperatively), and 11 were not related to exercise, including knee pain (*n* = 5), chest pain (*n* = 1), foot pain (*n* = 1), and other (*n* = 4) (10 preoperatively, 1 postoperatively). While we did not report analgesic regimens, standard of care included pain control with medications, ice packs, and positioning of the leg. We also acknowledge that reporting pain-specific sub-scores could enhance interpretability and appreciate the suggestion for future analyses.

We appreciate the commentary on our use of the 6-week postoperative time point for the study outcome measures. In our study, postoperative follow-ups were conducted at 6 weeks and 6 months, with preoperative assessments completed at baseline and 1 week prior to surgery, based on standard surgical assessment time points. While the reviewers suggested including cognitive and functional assessments such as the Timed Up and Go (TUG) and Montreal Cognitive Assessment (MoCA), our study instead measured the Short Physical Performance Battery (SPPB) and the Mini-Cognitive Assessment (Mini-Cog) to assess physical function and cognition, respectively. The SPPB is recommended by the European Working Group on Sarcopenia in Older People (EWGSOP) to measure physical performance[3], and is a key measure used to identify frailty in older adults in clinical practice and research. The Mini-Cog was not included in the current manuscript but will be incorporated in secondary analyses. It was selected over the MoCA as it takes only 3–4 min to administer, making it more feasible for use in the perioperative screening clinic compared to the MoCA, which can take up to 10 min. The Mini-Cog also demonstrates high sensitivity (73 %) and specificity (84 %)[4] to detect either mild cognitive impairment, dementia, or cognitive impairment, and is less influenced by age or education level. We also aimed to limit the number of questionnaires to reduce respondent burden. We agree that further cognitive testing is needed if functional issues are identified, and that assessing persistent cognitive impairment, delirium and functional decline is critical, particularly in frail older adults undergoing surgery who are at greater risk of adverse outcomes postoperatively[5].

Lastly, we agree that multimorbidity is a key determinant of both frailty and surgical outcomes. While we collected data on comorbidities such as cardiovascular disease and diabetes, some Charlson Comorbidity Index (CCI) items were not captured (e.g., solid tumour, lymphoma, Acquired Immunodeficiency Syndrome [AIDS]), limiting our ability to calculate comprehensive CCI scores. Nonetheless, we appreciate the proposal to stratify analyses by comorbidity burden, and will consider secondary analyses using available diagnostic data to examine the role of multimorbidity – potentially with the use of other comorbidity indices – on participant outcomes.

We appreciate the authors’ thoughtful engagement and their valuable suggestions to inform future study designs and analyses. The feedback offered will aid in our ongoing efforts to optimize perioperative care for frail older adults undergoing joint replacement surgery.

## Declarations

### Declaration of generative AI and AI-assisted technologies in the writing process

No generative AI or AI-assisted technologies have been used in the writing process.

## CRediT authorship contribution statement

**Alexandra Papaioannou:** Conceptualization, Methodology, Writing – original draft, Writing – review & editing. **Ashlee Azizudin:** Conceptualization, Methodology, Writing – original draft, Writing – review & editing. **George Ioannidis:** Conceptualization, Methodology, Writing – original draft, Writing – review & editing.

## Declaration of competing interest

The authors declare that they have no known competing financial interests or personal relationships that could have appeared to influence the work reported in this paper.

## References

[1] Okpara C., Negm A., Adachi J.D., Armstrong D., Atkinson S., Avram V. (2025). Getting fit for hip and knee replacement: the Fit-Joints multimodal intervention for frail patients with osteoarthritis – a pilot randomized controlled trial. J Frailty Aging.

[2] Dawson J., Fitzpatrick R., Murray D., Carr A. (1998). Questionnaire on the perceptions of patients about total knee replacement. J Bone Joint Surgery British.

[3] Cruz-Jentoft A.J., Bahat G., Bauer J., Boirie Y., Bruyère O., Cederholm T. (2019). Sarcopenia: revised European consensus on definition and diagnosis. Age Ageing.

[4] Abayomi S.N., Sritharan P., Yan E., Saripella A., Alhamdah Y., Englesakis M., Sakurai R. (2024). The diagnostic accuracy of the mini-cog screening tool for the detection of cognitive impairment—A systematic review and meta-analysis.

[5] Lin H.S., Watts J.N., Peel N.M., Hubbard R.E. (2016). Frailty and post-operative outcomes in older surgical patients: a systematic review. BMC Geriatr.

